# Liposomes Coated with Novel Synthetic Bifunctional Chitosan Derivatives as Potential Carriers of Anticancer Drugs

**DOI:** 10.3390/pharmaceutics16030319

**Published:** 2024-02-24

**Authors:** Elisabetta Mazzotta, Antonia Marazioti, Spyridon Mourtas, Rita Muzzalupo, Sophia G. Antimisiaris

**Affiliations:** 1 Department of Pharmacy, Health and Nutritional Sciences, University of Calabria, Via Savinio, Ed. Polifunzionale, 87036 Arcavacata di Rende, Italy; rita.muzzalupo@unical.it; 2 Laboratory of Pharmaceutical Technology, Department of Pharmacy, University of Patras, 26510 Rio Patras, Greece; a.marazioti@uop.gr (A.M.); mourtas@upatras.gr (S.M.); santimis@upatras.gr (S.G.A.); 3 Laboratory of Basic Sciences, Department of Physiotherapy, School of Health Sciences, University of Peloponnese, 23100 Sparta, Greece; 4 Department of Chemistry, University of Patras, 26504 Rio Patras, Greece; 5 Foundation for Research and Technology Hellas, Institute of Chemical Engineering Sciences, FORTH/ICE-HT, 26504 Rio Patras, Greece

**Keywords:** liposomes, polymer coating, stimuli-responsive, multifunctional polymers, cancer therapy

## Abstract

In this study, liposomes coated with novel multifunctional polymers were proposed as an innovative platform for tumor targeted drug delivery. Novel Folic acid–Cysteine-Thiolated chitosan (FTC) derivatives possessing active targeting ability and redox responsivity were synthesized, characterized, and employed to develop FTC-coated liposomes. Liposomes were characterized for size, surface charge and drug encapsulation efficiency before and after coating. The formation of a coating layer on liposomal surface was confirmed by the slight increase in particle size and by zeta-potential changes. FTC-coated liposomes showed a redox-dependent drug release profile: good stability at physiological conditions and rapid release of liposome-entrapped calcein in presence of glutathione. Moreover, the uptake and cytotoxic activity of doxorubicin-loaded FTC-coated liposomes was evaluated on murine B16-F10 and human SKMEL2 melanoma cancer cells. Results demonstrated enhanced uptake and antitumor efficacy of FTC-coated liposomes compared to control chitosan-coated liposomes in both cancer lines, which is attributed to higher cellular uptake via folate receptor-mediated endocytosis and to triggered drug release by the reductive microenvironment of tumor cells. The proposed novel liposomes show great potential as nanocarriers for targeted therapy of cancer.

## 1. Introduction

Multi-target drug delivery systems are innovative pharmaceutical devices emerging as one of the most promising challenges in cancer therapy. The major limitation of traditional chemotherapy is low drug selectivity. The use of supramolecular carriers aiming to modify drug distribution and to improve pharmacokinetic/pharmacodynamic drug profiles has been extensively reported in the literature [1]. Several approaches such as active and passive targeting have been, indeed, studied to achieve controlled drug release, increased cellular uptake, enhanced drug distribution, prolonged half-life and reduced side effects. However, the results acquired to date are far from optimal and inadequate to obtain clinical efficacy. To further improve drug performance, the design of tailor-made carriers combining several targeting approaches in a single system has been actively pursued in the last years. Smart engineered nanocarriers exhibiting multiple tumor targeting properties simultaneously have shown great promise in clinic and could be used in the near future as tools for personalized cancer therapy. Different strategic combinations have demonstrated high control of drug release at the site of action and reduced drug leakage, avoiding toxic effects on healthy cells [2,3].

One of the strategies adopted to increase tumor targeting capability is the design of stimuli-responsive nanodevices. Both biological/endogenous and non-biological/exogenous stimuli are currently investigated as triggers to activate a fast and localized drug release within the interested target site [4]. External stimuli-responsive nanoparticles offer some advantages including spatial, temporal and dose control of drug release through a remote apparatus that can be switched on and off, at will [5]. Common external signals used are temperature [6], magnetic field [7,8], ultrasound [9] and light [10]. On the other hand, drug release can also be triggered by specific biological signals of altered cancer cell microenvironment. Tumors normally present low pH [11], high redox potential [12] and specific proteolytic enzymes [13] and, thus, carriers can be smartly engineered to undergo rapid changes in their structure only under exposure to such conditions. Among the stimuli investigated for anticancer drug delivery, very interesting results have been achieved with glutathione (L-γ-glutamyl-L-cysteinylglycine, GSH), a natural antioxidant involved in cellular redox homeostasis [14]. GSH is the major organic reducing agent in the human body protecting the cells from harmful effects of free radicals. Due to the role of GSH in tumor initiation, progression and drug resistance [15,16], GSH concentrations inside cancer cells (2–20 mM) are, in fact, significantly higher (thousand times higher) than those in the extracellular matrix (2–20 µM) or in normal healthy cells [5].

Therefore, its incorporation in nanocarriers with disulfide linkages that are cleavable by GSH is an ongoing promising approach to obtain selective and localized drug release. Notably, disulfide bonds act as controlled gates, thus preventing premature drug leakage, decreasing the systemic toxicity and triggering the release only in the redox-rich environment of cancer cells [17].

Moreover, active targeting approaches exhibit unique advantages in current cancer therapy. This strategy refers to a selective molecular recognition between ligands that are immobilized on the carrier surface and specific cellular receptors located on tumor tissues. Many cancer cells, indeed, overexpress specific receptors in response to their increased metabolic demand. Specific interaction of such modified carriers with cell receptors results in enhanced carrier accumulation in the tumor microenvironment and, subsequently, increased internalization via receptor-mediated endocytosis. A large variety of molecules such as antibodies, proteins, cell penetrating peptides, sugars and small molecules have been extensively investigated for active targeting of drug-loaded carriers to cancer tissues [18]. Folic acid (FA) is one of the most popular approaches because its receptors are overexpressed in a wide range of epithelial cancer cells such as ovary, breast, liver, brain, colon, lung and kidney [19], exhibiting high binding affinity (dissociation constant (Kd) < 1 nM) [20]. On the contrary, the folate receptor has low expression frequency in normal healthy cells. Folate receptors actively internalize folate conjugated devices via receptor-mediated endocytosis. Consequently, surface functionalization of nanocarriers with folic acid represents an attractive strategy to increase the uptake and accumulation of chemotherapeutic drugs at tumor sites.

Liposomes are nanovesicular systems suitable for the design of multifunctional devices due to their excellent features such as biocompatibility, biodegradability, sustained and controlled release [21,22,23]. Their application in cancer treatment is still limited by various drawbacks related to premature drug leakage, low physical and chemical stability and non-specific targeting [24,25]. Surface coating of liposomes with biocompatible polymers has been proposed as a potential way to overcome some of these limitations [26,27]. In particular, the coating of liposomes with multi-targeted polymers may be a strategic approach aiming to enhance target specificity towards cancer. A recent study focused on the design of liposomes coated with pH-sensitive polymers and demonstrated enhanced anticancer activity and cellular uptake of such vesicles [28]. 

Taking into account all the considerations mentioned above, we developed herein a novel type of liposomes which are coated with a novel polymer that combines active targeting and redox responsivity as an innovative platform carrier for intracellular anticancer drug delivery. Chitosan (CHT), a natural, biocompatible polymer widely used in the design of pharmaceutical carriers, was selected in this study due to its non-toxicity, biocompatibility, mucoadhesive properties and versatility. The presence of a wide range of functional chemical groups in CHT’s structure makes it possible to be functionalized with various molecules, capable of conferring specific properties such as targeting ability, stimuli responsivity and improved solubility [29]. In fact, CHT was previously functionalized with targeting agent FA and L-cysteine (L-Cys) which can be easily oxidized by air to give inter- and intramolecular disulfide bonds. Nanoparticles that consist of this multifunctional polymer exhibited targeted and redox-responsive release of loaded compounds, resulting in an enhanced anticancer activity compared to the corresponding non-targeted nanoparticles [30]. Herein, we decided to use the same multifunctional polymer to coat liposomes and to evaluate whether this coating confers to liposome cancer-targeting properties. Indeed, liposomes are already used in clinical settings, and the coating process allows the use of considerably smaller quantities of the polymer with cancer target properties. Therefore, this strategy could represent an attractive advantage for translating the developed systems into a product to be used in clinical therapy. 

After confirming the sensitivity of the developed vesicles to reduced environment, doxorubicin (Dox), an anthracycline antibiotic with broad-spectrum antitumor activity, was loaded in liposomes and the cytotoxicity of the liposomes coated with the new conjugate was investigated in B16-F10 and SKMEL2, murine and human melanoma cancer cell lines, respectively. The uptake of the various liposome types by the same cells was additionally studied.

## 2. Materials and Methods

### 2.1. Chemicals 

1,2-distearoyl-*sn*-glycerol-3-phosphatidylcholine (PC) and 1,2-distearoyl-*sn*-glycero-3-phospho-(19-rac-glycerol) (sodium salt) (PG) were purchased from Lipoid (Ludwigshafen, Germany). Chitosan (low molecular weight), N-cyclohexyl-N′-(2-morpholinoethyl)carbodiimide-metho-p-toluenesulfonate, L-cysteine, folic acid, Cholesterol (Chol), chloroform, doxorubicin hydrochloride, calcein, fluorescein–isothiocyanate–dextran-4000 (FITC), glutathione, Triton X-100, and 3-[4,5-dimethylthiazol-2-yl]-2,5 diphenyl tetrazolium bromide were purchased from Sigma-Aldrich (Milano, Italy). Doxorubicin Hydrochloride (Dox) was obtained from Tocris (Bristol, UK). The RPMI medium, fetal bovine serum (FBS), Trypsin–EDTA and all other cell culture solutions used were purchased from Gibco (Monza, Italy). The organic solvents used were supplied by VWR International SRL (Milan, Italy) and Sigma-Aldrich. Fluorescence intensity (FI) of samples was measured by a Shimatzu RF-1501 spectrofluoremeter (Shimatzu, Kyoto, Japan).

### 2.2. Preparation of Folic-L-Cys-Thiolated-Chitosan

Folic acid–L-cysteine-thiolated chitosan (FTC) conjugates were synthesized through a two-step process. Firstly, the conjugation of L-Cys to the chitosan backbone was achieved via amide formation using the unprotected L-Cys. Three different thiolated chitosan (TC) derivatives were synthesized, and the quantities used are reported in Table 1. Briefly, the carboxylic acid group of L-Cys was activated by N-cyclohexyl-N′-(2-morpholinoethyl)carbodiimide-metho-p-toluenesulfonate (CMCT) in deionized water for 1 h. Next, CHT was dissolved in acetic acid 1% (*v*/*v*) to obtain a 1% (*w*/*v*) polymer solution and this was added dropwise to the above solution under magnetic stirring. The pH of the reaction mixtures was carefully adjusted to 5.0 with NaOH 0.5 M. After that, the mixture was reacted in the dark at room temperature under stirring for 4 h. The resulting solution was extensively dialyzed [molecular weight (MW) cutoff 12 kDa] for 3 days, first against 5 mM HCl, twice against 5 mM HCl containing 1% NaCl, and finally against 1 mM HCl. Finally, the polymer solutions were lyophilized and used for the synthesis of folic acid-thiolated chitosan (FTC).

In the second step, CMCT (0.04 mmol) was added to 2 mg/mL of FA (0.02 mmol) in dimethyl sulfoxide and stirred at room temperature for 1 h. Then, 100 mg of TC was dissolved in 10 mL of acetic acid 1% (*v*/*v*), added to the above solution and stirred in the dark at room temperature for 16 h. The mixture was thus precipitated leading the pH to 9. The flocculent precipitate was collected and then dialyzed (molecular weight cut-off of 12 kDa) against an excess amount of 0.1 M sodium phosphate buffer (pH 7.4) for 3 days and then against water for 3 days. Finally, the resulting mixture was freeze-dried, and the resulting products were obtained and stored at 4 °C until further use.

### 2.3. Characterization of FTC Conjugates

#### 2.3.1. H-NMR Analysis

NMR spectra were recorded on a Brucker DPX 600 MHz instrument (Peoria, IL, USA). The sample spectra were recorded at 25 °C (chemical shifts (δ) were referenced to the corresponding solvent peaks and are reported in parts per million (ppm)). The samples were prepared as follows: chitosan and the three FTC polymers were dissolved in deuterium oxide (D_2_O) and acidified with 2 % (*v*/*v*) trifluoroacetic acid (CF_3_COOH).

#### 2.3.2. Quantification of Folic Acid

The amount of FA conjugated on CHT was evaluated using UV–Vis spectroscopy, considering its absorption at 285 nm. The conjugates were dissolved in 1% acetic acid at the concentration of 2 × 10^−2^ mg/mL and the measurements of the absorbance of these solutions were performed. FA concentration was calculated on the basis of a standard curve previously plotted from a stock solution of FA dissolved in a 0.1 M NaOH solution, from which subsequently dilutions in 1% acetic acid were prepared in the range of 0.2–2 × 10^−2^ mg/mL.

#### 2.3.3. Determination of Thiol and Disulfide Bond Content

The amount of thiol groups on the polymers was determined via iodometric titration [31]. Firstly, a storage iodine solution was prepared at concentration of 0.1 M by dissolving 0.63 g of I_2_ and 1.93 g of KI in 25 mL of distilled water and diluted just before the assay. Each polymer was hydrated in 0.5 M acetate buffer, pH 2.7, in different concentrations (0.5–0.05 mg/mL). Then, 1 mL of starch aqueous solution (1%, *w*/*v*) and 1 mL of iodine (1 mmol/L) were added to 2 mL of each sample. Iodine promotes the oxidation of free thiol groups. The excess of iodine reacts with starch, causing a blue complex which is easily measured at 560 nm. The amount of thiol moieties was calculated from a standard curve of L-Cys in a concentration range of 20–70 mol/L. Disulfide bond content was measured after reduction with sodium borohydride (NaBH_4_) and conversion to the corresponding reduced thiol groups. Control samples were elaborated with non-thiolated chitosan.

### 2.4. Liposome Preparation

Multilamellar (MLV) and small unilamellar (SUV) liposomes made up of PC/PG/Chol, 9:1:5 (mol/mol/mol) were prepared by the thin film hydration technique as reported previously. In brief, accurately weighted amounts of lipid were dissolved in a chloroform/methanol (2:1 *v*/*v*) mixture in a round-bottom flask. The solvents were evaporated under reduced pressure and constant rotation to form a thin lipid film on the walls of the flask. Hydration of the lipid film was performed by adding 1 mL of the PBS buffer, pH 7.4, at 42 °C and applying bath sonication and intensive vortex. In some cases (for vesicle/cell interaction studies), FITC, 36 mM in PBS, was used for lipid film hydration. The obtained liposome dispersion was thus left to equilibrate for 2 h at a temperature above the transition temperature of the lipid (42 °C) in order to allow complete annealing of any structural defects. SUVs were prepared from MLVs applying probe sonication until the formation of a clear dispersion. Titanium fragments released from the probe as result of the high intensity of sonication were removed by centrifugation at 15,000 rpm for 20 min. Finally, the liposome dispersions were either stored at 4 °C or used immediately. When liposomes were loaded with FITC, they were purified from non-encapsulated dye by ultracentrifugation (40,000 rpm for 1 h) and then coated (when applying) as described below.

### 2.5. FTC Coating of Liposomes

The coating of liposomes was performed using a polymer/lipid mass ratio equal to 0.1, reported as the best condition to achieve the maximum coating efficiency in a previous work [32]. Firstly, coating solutions were prepared dissolving the polymers in isotonic acetate buffer (pH 4.4). The exact phospholipid content of the liposomes was measured by the Stewart assay in order to identify the concentration of the polymer solution which was necessary to achieve FTC/PC equal to 0.1 (mass ratio). The polymer solution was added dropwise into the liposome dispersion at an equal volume, under continuous stirring for 1 h. CHT-coated liposomes were prepared in the same conditions as control.

The coating efficiency (CE%) of the liposomes was determined as the ratio between the phospholipid content within the FTC-coated liposomal fraction and the total phospholipid content of liposomes. For this, the FTC-coated liposomes were subjected to centrifugation at 14,000 rpm for 20 min until complete sedimentation of the coated liposomes. The phospholipid concentration before and after centrifugation was determined by the Stewart assay. Thus, CE % was calculated using the following equation:

$$\mathrm{CE}\%=\frac{\mathrm{lipid}\;\mathrm{content}\;\mathrm{in}\;\mathrm{pellet}}{\mathrm{lipid}\;\mathrm{content}\;\mathrm{before}\;\mathrm{the}\;\mathrm{centrifugation}}\times 100.$$

### 2.6. Liposome Physicochemical Characterization

#### 2.6.1. Stewart Assay

The lipid concentration of liposome dispersions was measured by the Stewart assay [33] based on the formation of a colored complex between phospholipids and ammonium ferrothiocyanate (485 nm). Firstly, the Stewart reagent was prepared by dissolving 27.03 g of FeCl_3_ 6 H_2_O and 30.4 g of ammonium thiocyanate in 1 L of double distilled water. Then, 20 μL of each liposomal suspension were mixed with 2 mL of the Stewart reagent and 2 mL of chloroform. The resulting mixture was intensively vortexed for extraction of the lipid complex in the organic phase and, after that, centrifuged at 4000 rpm for 5 min. The lipid concentration was calculated on the basis of a standard curve prepared with a known amount of each phospholipid. 

#### 2.6.2. Size Distribution and Zeta Potential Measurements

Liposome size and distribution before and after coating were measured by the dynamic light scattering technique (Malvern Nano-Zs; Malvern Instrument, Malvern, UK) at 25 °C and an angle of 173°. The Polydispersity Index (P.I.) was used as a measure of size distribution. The P.I. of less than 0.3 indicates a homogenous and monodisperse population in the case of colloidal systems [34]. Zeta potential was also measured at 25 °C by the same instrument using the Doppler electrophoresis technique. 

### 2.7. In Vitro Redox-Responsive Release Studies 

To evaluate the redox-responsive nature of the designed liposomes, the release experiments were performed in presence or absence of GSH in order to simulate the intracellular compartment of cancer cells and the physiological environments. Calcein, a water soluble and fluorescent probe, was used as a release molecule and calcein-loaded SUV liposomes were initially prepared as described above using a buffered solution containing calcein in a quenched concentration (100 mM). Calcein liposomes were separated from non-entrapped calcein by gel filtration on a sephadex G-50 (1 cm × 30 cm) column eluted with a PBS buffer. Subsequently, the liposomes were coated using a 0.1 (*w*/*w*) FTC/lipid ratio by the method mentioned above. CHT-coated liposomes were also prepared, and their release profiles were evaluated in the same conditions.

Aliquots of liposome suspension were placed in dialysis bags (0.5 mL), manipulated before use (Visking dialysis tubes, 20/30 cut-off: 12,000–14,000 Da) and suspended in 20 mL of phosphate buffer saline (PBS, pH 7.4) with or without 10 mM GSH at 37 °C. At prescribed time intervals, 2 mL of the release medium were withdrawn maintaining the volume of the receptor compartment with an equal volume of fresh solution. The total amount of calcein in the withdrawn samples was determined by the quantitative fluorescence spectrophotometric method (emission wavelength: 470 nm, excitation wavelength: 520 nm). The results are presented in terms of cumulative release as a function of time. All calcein release studies were carried out in triplicate. 

### 2.8. Doxorubicin Liposome Preparation

Dox was loaded into the liposomes using the active loading technique, as previously described [7]. According to this method, PC/PG/Chol liposomes were prepared in ammonium sulfate ([NH_4_]_2_SO_4_, 120 mM) instead of PBS, as described above. The un-trapped external ammonium sulfate was removed by ultracentrifugation at 40,000 rpm for 2 h, and then the pellet was resuspended in a PBS, pH 7.4. Subsequently, a Dox solution (0.2 mg/mL) was added to the liposomes at a 7:1 phospholipid/Dox weight ratio and incubated at 60 °C for 1 h protected from the light. Finally, the liposomes were first purified from non-encapsulated drug by ultracentrifugation (40,000 rpm for 1 h) and then coated as described above.

Dox encapsulation efficiency (EE%) in liposomes was calculated by measuring the molar ratio of drug over lipid [D/L (μmol/μmol)] before and after liposome purification (from non-encapsulated drugs) according to the following equation:

$$\mathrm{EE}\left(\%\right)=\frac{[D/L(\mu \mathrm{mol}/\mu \mathrm{mol})]\;\mathrm{final}}{[D/L(\mu \mathrm{mol}/\mu \mathrm{mol})]\;\mathrm{initial}}\times 100$$

Additionally, the loading capacity of Dox in liposomes was calculated according to the following equation:

$$\mathrm{Loading}\;\mathrm{capacity}\left(\%\right)=\frac{\mathrm{mass}\;\mathrm{of}\;\mathrm{loaded}\;\mathrm{drug}}{\mathrm{mass}\;\mathrm{of}\;\mathrm{drug}\;\mathrm{loaded}-\mathrm{liposomes}}\times 100$$

EE% and loading capacity% of the drug as well as the lipid content of each liposome preparation was measured as described above. 

For the measurement of Dox concentration, 200 µL of each liposome dispersion was completely dissolved in 1.98 mL of PBS and 2 mL of a 10% (*v*/*v*) solution of Triton X-100 (in H_2_O). Then, the absorption of Dox at a 481 nm wavelength was recorded on an UV–vis spectrometer and the concentration was calculated according to the standard curve constructed by standard solutions of Dox in PBS/Triton 10% (linear in the 2.5–40 ppm range). 

For measurement of phospholipid concentration of liposomes, the Stewart assay was used [33].

### 2.9. In Vitro Cytotoxicity Assay 

The cytotoxic effects of empty and Dox-loaded liposomes coated with new polymers were evaluated towards B16-F10 melanoma cancer cells and human melanoma SKMEL2 cells using the 3-[4,5-dimethylthiazol-2-yl]-2,5 diphenyl tetrazolium bromide (MTT) dye test (cell viability) The cells were grown in an RPMI medium (Gibco, Monza, Italy) supplemented with penicillin–streptomycin (100 mg/mL) and 10% heat-inactivated fetal bovine serum (FBS) at 37 °C, 5% CO_2_/saturated humidity. For the assay, the cells were seeded at a density of 5 × 10^4^ cells/mL in 24-well tissue culture plates for 24 h to allow the adhesion of the cells. The culture medium was then replaced with fresh medium and the Dox-loaded liposomes were added at three concentrations; 1, 3 and 6 μM (empty liposomes were added at the corresponding lipid concentrations). After 3 h, the cells were washed and incubated with fresh medium for another 45 h.

Untreated B16-F10 cells and SKMEL2 cells were used as controls in each corresponding study. At the end of the incubation time, 50 μL of MTT tetrazolium salt (5 mg/mL dissolved in PBS) were added to each well and the cells were incubated at 37 °C and 5% CO_2_ for an additional 4 h to allow the formation of violet formazan crystals. Acidified isopropanol (500 μL) was added to solubilize the formazan crystals in the cells. Viable cells (%) were calculated based on the formula (A570sample–A570background)/(A570control–A570background) × 100, where A570control is the OD-570 nm of untreated cells and A570background the OD-570 nm of MTT without cells. Values of cell viability are expressed as the mean of at least three different experiments ± S.D.

### 2.10. In Vitro Cell Uptake Experiments

In order to further evaluate the mechanism of potential increased cytotoxicity of the Dox-loaded liposomes coated with new polymers (compared to non-coated and CHT-coated liposomes), we sought to study the uptake of the various liposome types which were loaded with FITC, as described above.

For evaluation of the uptake of FITC-loaded liposomes by cells, FITC-LIPs (400 nmol liposomal lipid/10^6^ cells) were incubated with confluent monolayers of B16F10 and SKMEL2 cells, in RPMI medium (containing 10% FBS (*v*/*v*)) at 37 °C, for 1h and 4 h. After that, the medium was removed and the cells were washed twice with ice-cold PBS, detached from plates by scraping, re-suspended in 1 mL of PBS and assayed for FITC FI (EX-490 nm/EM-525 nm, 5 nm slits) after cell lysis in 2% Triton X-100. Cell autofluorescence was very low and was always subtracted. The protein content of all samples was measured by the Bradford assay, and DOX uptake by cells was normalized to the cellular protein concentration of each sample.

### 2.11. Statistical Analysis

Data are expressed as mean ± S.D. of at least three independent experiments. Statistical analysis was performed using Student’s *t*-test. *p*-values ≤ 0.05 were considered statistically significant.

## 3. Results

### 3.1. Synthesis and Characterization of FTC Conjugates

**Figure 1:**
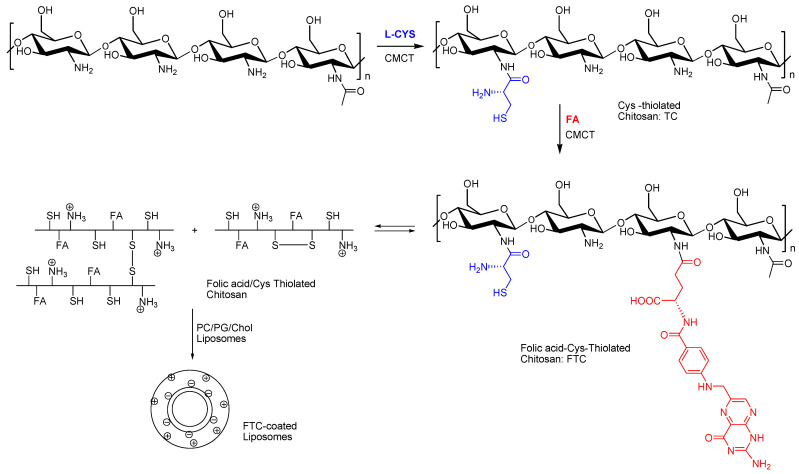
Synthetic route for folic-thiolated chitosan (FTC). Abbreviations: L-cysteine (L-CYS); N-cyclohexyl-N′-(2-morpholinoethyl)carbodiimide-metho-p-toluenesulfonate (CMCT); folic acid (FA).

In this study, novel folate redox-responsive chitosan derivatives were designed, synthesized, and characterized. The aim was to combine redox responsivity and active targeting into a single polymer for improved specificity towards targeted cancer cells. FTC conjugates were synthetized in a two-step synthetic approach by covalent coupling of L-Cys and FA to CHT using carbodiimide chemistry, as shown in Figure 1. In brief, L-Cys was activated with N-cyclohexyl-N′-(2-morpholinoethyl)carbodiimide-metho-p-toluenesulfonate (CMCT), and then reacted with CHT to afford the thiolated chitosan (TC) conjugate. This was further reacted with activated FA to afford the desired FTC (details for the synthetic procedure can be found in the Materials and Methods section). 

In general, CHT is dissolved in 1% acetic acid and the first coupling reaction is performed at pH = 5.0, where CHT equilibrates between its protonated and unprotonated form (pKa (NH_2_) = 6.3–6.5), allowing the conjugation of Cys to the CHT backbone, while at the same time the amine group of Cys is almost exclusively protonated (pKa (NH_2_) = 10.78). In addition, thioester bonds that may have been formed during the activation of L-Cys, though the reaction of the thiol group of Cys with activated carboxylic acid groups, are expected to also react with CHT amine groups to afford the desired CHT-Cys covalent amide bond. The same picture applies for the second step of conjugation, where TC is also dissolved in 1% acetic acid and the pH of the reaction mixture directs regioselectivity again by the reaction of CHT amine groups with the activated γ-carboxylic acid groups of FA. Air oxidation of the sulfhydryl groups during reaction is prevented by performing the whole process at low pH, where the concentration of thiolate anions is low (side chain pKa = 8.37) and the formation of the disulfide bond is slower. Nevertheless, some oxidation of the free thiol groups of FTC polymers to the corresponding disulfides by inter and intra-molecular disulfide bond formation is expected during the whole procedure [35]. The expected product(s) from this simplified method of sequential L-Cys thiolation and FA conjugation to chitosan is (are) presented in Figure 1. 

In order to characterize the chemical structure(s) of the synthesized bifunctional products, ^1^H-NMR and UV–vis spectroscopy were performed.

**Figure 2:**
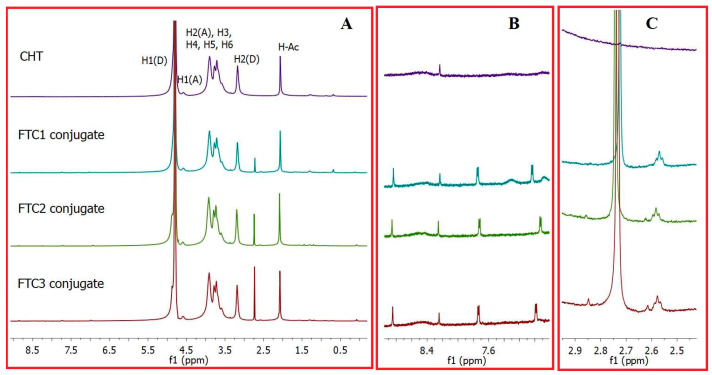
(**A**) ^1^H-NMR spectra of the obtained FTC1, FTC2, FTC3 conjugates in comparison with CHT (A: acetylated; D: deacetylated; Ac: acetyl group); (**B**) Enlarged ^1^H-NMR profile in the region of 8.6-6.9 ppm; (**C**) Enlarged 1H-NMR profile in the region of 2.9–2.5 ppm.

In brief, the ^1^H-NMR spectra of the FTC conjugates showed a new peak at 2.73, which was assigned to methylene protons next to the thiol group. This peak, although slightly shifted due to the expected adjacent disulfide bond formation when recording, confirms the successful conjugation of L-Cys to the CHT backbone (Figure 2) [36]. As can be seen, this peak is not present in the ^1^H-NMR of CTH, while it appears in the spectrum of FTC1, FTC2 and FTC3 conjugates (Figure 2). In addition, the successful conjugation is also confirmed by the increasing ratio of this peak from FTC1 to FTC3, where increased amounts of Cys are expected to be conjugated. Moreover, the appearance of peaks at 8.83 ppm (s, H-C=N pterin ring), 7.74 ppm (d, H-Ar p-amino benzoic acid ring), 7.03 ppm (d, H-Ar p-aminobenzoic acid ring), which correspond to the aromatic ring of the folate, and the triplet at 2.57 ppm, which was assigned to γ-methylene protons of glutamic acid (which is part of folic acid molecule), reveals the effective incorporation of this targeting molecule on the CHT backbone. The successful FA conjugation was further confirmed by UV–vis spectroscopy. In fact, the typical absorption peak of FA at 280 nm was clearly observed and the content of conjugated FA was measured to be 59.70 ± 4.60, 28.16 ± 1.04 and 45.85 ± 4.92 μmol/g polymer, respectively, for FTC1, FTC2 and FTC3. Iodometric titration was used to measure the free thiol groups and disulfide bond content in the polymers, and the results of quantitative assay are reported in Table 2. As can be seen, the highest degree of conjugation was achieved using a polymer/L-Cys ratio of 1:4. Moreover, the 41.13%, 33.61% and 62.00% of the total amount of free thiol groups were oxidized to the corresponding disulfides possibly during the conjugation or purification process of FTC1, FTC2 and FTC3, respectively (Table 3). Besides the presence of oxygen, and the fact that the coupling of Cys was performed at pH = 5.0, the coupling of FA required the use of DMSO, which is known to oxidize free thiols to dithiols and allows faster generation of dynamic combinatorial libraries of the corresponding disulfides over a wide pH range [37,38].

### 3.2. Characterization of Liposomes

Redox-responsive nanocarriers were obtained by coating traditional liposomes made up of PC/PG/Chol with the multi-functional polymers synthesized. The physical polymer coating can offer some attractive features to liposomes, such as improved stability, accompanied by prolonged circulation and resistance to macrophage uptake, redox responsivity and active targeting properties. Several studies were carried out in order to confirm the effective formation of a coating layer on the liposome surface and to evaluate its effect on vesicle physicochemical characteristics, since there are key aspects that contribute to the overall behavior of the systems in vitro and in vivo.

The mean diameters, polydispersity index (PI) and zeta potential of liposomes before and after coating are given in Table 3. The designed liposomes showed a specific distribution size depending on the presence or absence of the coating layer. In fact, uncoated liposomes had a mean size range between 57.35 nm and 134.10 nm with a PI always ≤ 0.3 indicating a narrow size distribution. The coating process, as expected, induced a slight increase in the vesicle size without negatively affecting the characteristics of the systems. The designed liposomes had an average diameter between 83.12 and 225.4 nm as can be seen in Table 3. This small size is a desirable feature for anticancer delivery systems since it is a crucial parameter in influencing their stability in vivo. Commonly, liposomes with small size are associated to extended bioavailability and higher tumor accumulation through the EPR effect compared to larger liposomal formulations [13].

An interesting, highly repeatable observation was that the mean diameter of liposomes coated with plain CHT was higher than that of the FTC-coated ones. This could be explained by the formation of intermolecular disulfide bonds among thiol groups located close to different polymer chains in the new conjugate [39]. These intermolecular linkages obviously allow the development of a denser and compact structure, decreasing the size of the FTC–liposome structure that is finally formed. 

The presence of a polymer coating on liposome surfaces was also confirmed by the inversion of the zeta potential from negative to positive values. Uncoated liposomes showed a zeta potential equal to −20.9 ± 1.1mV, while polymer-coated liposomes had a zeta potential ranging between +27.8 ± 0.4 and +31.2 ± 0.1 mV. The shift in the zeta potential towards positive values can be attributed to the positive nature of the polymers employed, which indicates successful conjugation on the liposome surface. The formation of the coating layer is, indeed, based on the electrostatic interaction between the positively charged polymer chains and the negative head groups of phospholipids. In these colloidal systems, the large positive zeta-potential values are also indicative of high electrostatic repulsion forces between particles, thus leading to high stability of the designed systems, with no appearance of sedimentation and/or coagulation within 3 months.

Moreover, the positive nature of the designed liposomes may improve their vascular targeting ability and tumor accumulation due to electrostatic interactions with anionic molecules in the tumor microvasculature, such as proteoglycans, glycoproteins and anionic phospholipids [40].

**Figure 3:**
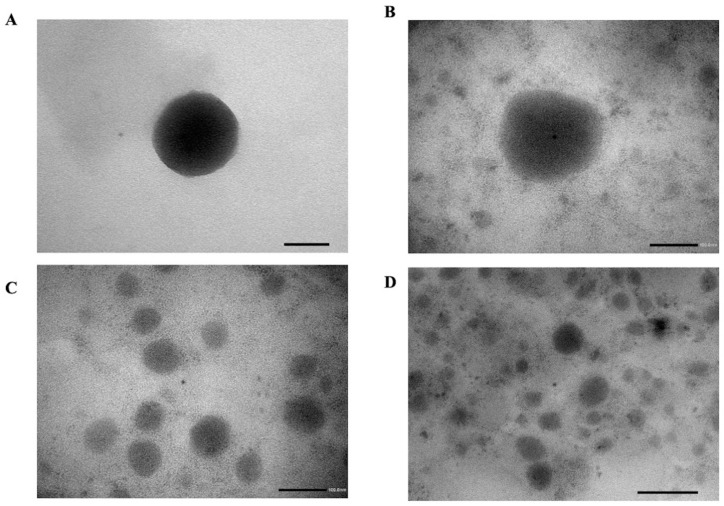
Typical photomicrographs of (**A**) non-coated liposomes (scale bar 50 nm) and (**B**,**C**) FTC2-coated liposomes (scale bar 100 nm) and (**D**) liposomes (scale bar 200 nm).

TEM analysis was carried out to visualize the morphology of uncoated and coated liposomes (Figure 3). The images indicated that FTC-coated and uncoated liposomes were nanosized and have a spherical shape with no significant morphological differences indicating that the FTC polymer did not alter the nanoparticle structure. 

The coated polymer over liposome ratio is a key parameter to evaluate coating ability. The CE% values measured for PC/PG/Chol liposomes coated using CHT resulted in 77.58%, in good agreement to previous study [31]. In the case of new conjugates, the CE% was found to be 94.39%, 91.23% and 88.47%, respectively, for FTC1, FTC2 and FTC3 indicating a better coating ability compared to CHT.

### 3.3. In Vitro Release Studies

**Figure 4:**
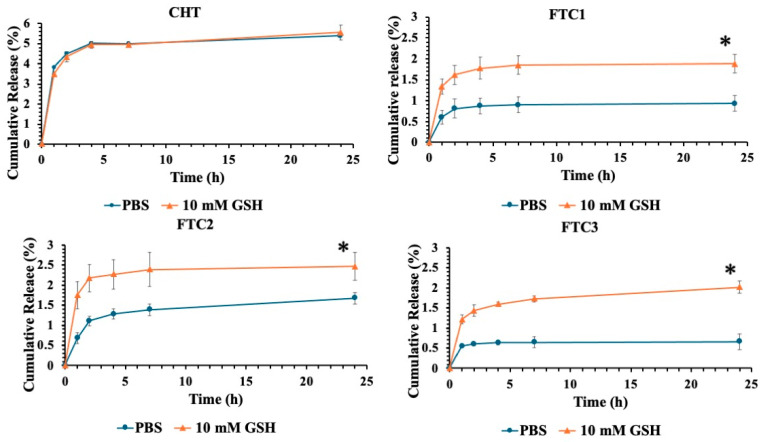
Redox-triggered release of calcein from polymer-coated liposomes in phosphate buffer, pH 7.4, in the absence (●) and presence of GSH 10 mM (▲) at 37 °C (mean ± standard deviation, n = 3). CHT-coated liposomes were used as a reduction-insensitive control. In all cases, each value represents the mean ± S.D. of three independent experiments. At every sampling time, percentage of the drug released in the GSH 10 mM medium was statistically different (* *p* < 0.05) from that recorded in a medium without GSH.

Extended drug retention in blood circulation and triggered release at the target site are important parameters in order to develop an optimal intracellular drug delivery system. To this end, the physical coating of liposomes with polymers containing disulfide bonds was proposed in this work. The disulfide bonds on surface coating should be able to reduce drug leakage during blood circulation and allow a selective and quick release triggered by the presence of reducing agents in tumor microenvironment. We thus decided to perform release studies using GSH as a reducing agent. The experiments were carried out in PBS, pH 7.4, in the presence and absence of 10 mM of GSH considering the concentrations of reducing agents in the cancer cell cytosol and in the extracellular matrix/blood plasma, respectively. The release profiles of calcein used as a model molecule from FTC-coated liposomes are shown in Figure 4. It is clearly seen that the release rates were markedly influenced by GSH. The drug release at pH 7.4 was very low, indicating a high stability of disulfide bonds in the polymer backbone at physiological conditions. 

Particularly, the release rate from FTC-coated liposomes resulted be lower than that obtained with CHT-coated ones indicating the better stability of the developed formulations in the blood circulation. The presence of intermolecular disulfide linkages, indeed, protects the drug against premature leakage and maximizes its location on the action site of applications avoiding the occurrence of side effects. On the contrary, in presence of GSH, the trend was already different, since the first hour. Calcein release was significantly greater throughout the investigated time range in the applied reductive conditions, predicting a rapid release in the reducing environment of cancer cells. This faster and sustained drug release can be attributed to the reduction in disulfide linkages due to their sensitivity to GSH, allowing for a less dense and compact structure. Thus, these formulations satisfy basic requirements of redox-responsive drug delivery systems for cancer therapy: drug retention during circulation and promoted release in response to reductive stimuli.

In particular, the three different polymers showed a different ability to minimize drug release at physiological conditions. Drug release at pH 7.4 resulted to be inversely proportional to the amount of disulfide bonds present in the polymer chains. These results clearly suggest that FTC-coated liposomes may control drug release in a redox-responsive manner. A control experiment using liposomes coated with CHT with neither disulfide bonds nor FA ligands was also performed under similar conditions. Calcein release from control liposomes did not exhibit GSH dependence. In fact, similar release kinetics in presence and absence of GSH were obtained. The current results confirmed our expectations and suggest that the presence of disulfide linkages could make nanocarriers highly stable before reaching tumor site but allow a complete and accelerated release after uptake in the redox environment of cancer cells. 

### 3.4. Dox-Loaded Liposomes: Physicochemical Characterization and Anticancer Activity

At this point, we decided to evaluate the potential anticancer activity of these multifunctional liposomes by loading them with Dox, a potent anticancer agent. Firstly, the drug was loaded in liposomes by active loading and afterwards the coating procedure was performed on the Dox-loaded vesicles. 

The sizes of drug-loaded vesicles before and after coating were small enough to extravasate from the blood into the tumor interstitial space (Table 3).

**Figure 5:**
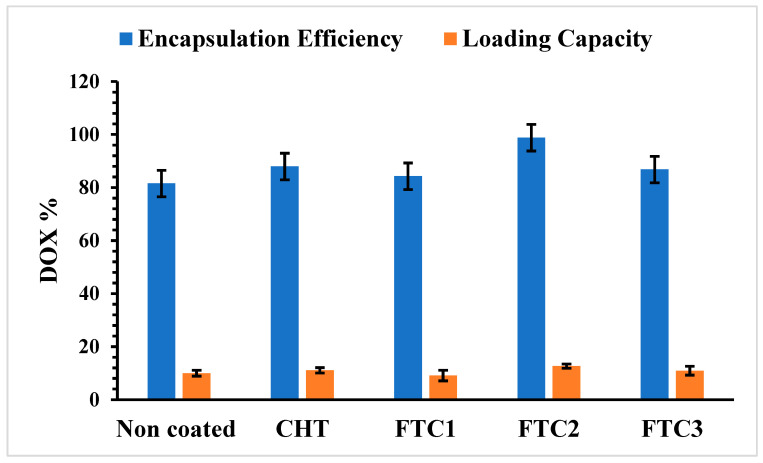
Dox encapsulation efficiency and loading capacity of uncoated and coated liposomes. Each result represent the mean value ± S.D. of three independent experiments.

The liposomes showed good capability in loading Dox, and no leakage of encapsulated Dox was observed during the coating process of both with CHT and FTC, as shown in Figure 5. On the contrary, higher drug entrapment efficiency was obtained in polymer-coated liposomes. This behavior was attributed to the compact layers on the liposomal surface which prevents any potential for drug loss [39].

### 3.5. In Vitro Evaluation of Coated Liposome Toxicity

**Figure 6:**
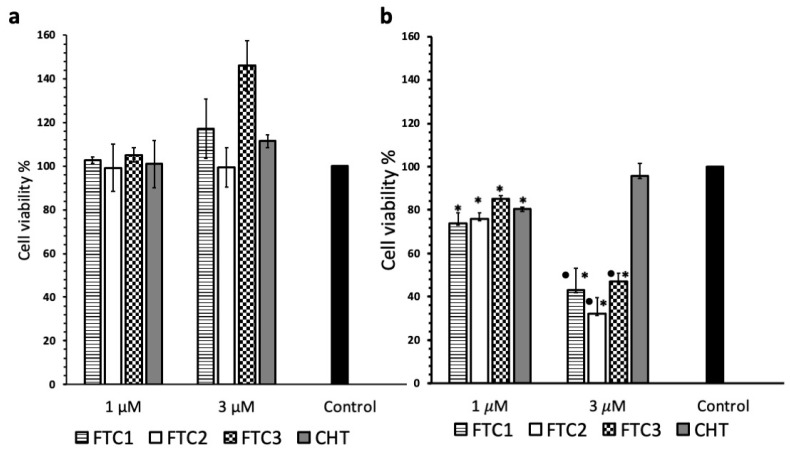
B16-F10 cell viability when exposed to empty (**a**) and Dox-loaded liposomes coated with different polymers (**b**) after 48 h at Dox concentrations of 1.0 and 3.0 μΜ. Cell proliferation is expressed as mean ± S.D. of three experiments and as the percentage of the control assumed to be 100%. * *p* < 0.05 vs. control; • *p* < 0.05 vs. CHT-coated liposome-treated cells.

B16-F10 and SKMEL2, murine and human melamona cells, respectively, were selected as models to evaluate the antitumor activity of Dox-loaded liposomes coated with FTC using non-target CHT-coated liposomes as control. In the first study, two different Dox concentrations, 1.0 and 3.0 μM, were used based on previous studies with the same cells that demonstrated reduced viability of B16-F10 cells at the corresponding Dox concentrations [41]. Initially, empty liposomes were evaluated in order to verify that the FTC coating does not confer any cytotoxicity to the vesicles. The results revealed that empty coated liposomes (both CHT and FTC) at the corresponding concentrations were practically non-toxic (the lowest cell viability measured was 88.3% at 48 h) under the conditions applied in the experiments in B16-F10 (Figure 6a). The developed empty FTC-coated liposomes showed a lower toxicity with respect to that obtained with nanoparticles made up only of FTC in a previous study [30], suggesting that the coating of the liposome surface is a promising strategy to develop multifunctional nanocarriers with reduced toxicity. Moreover, a significant increase in viability was observed for cells treated whit FTC1- and FTC2-coated empty liposomes. This effect could be due to the higher folic acid content of these polymers as reported above. In fact, recent studies reported that folic acid increased cell turnover and viability and decreased apoptosis in a dose-dependent manner [42,43].

On the contrary, a significant reduction in viability was observed in the cells exposed to liposomes coated with FTC and loaded with Dox (Figure 6b). In fact, although all the coated vesicles loaded with 1 μΜ Dox slightly reduced B16-F10 cell viability, there was no difference between the CHT-coated and the FTC-coated vesicles (all types). However, at the higher Dox concentration of 3 μΜ, a significant reduction in cell viability was observed for all FTC-coated liposomes compared to the ones coated with CHT alone. In detail, cell viability was slightly reduced (to 89.61% compared to the control) after treatment with CHT-coated liposomes, while the corresponding values obtained after treatment with FTC1, FTC2 and FTC3 liposomes were 42.99, 32.24 and 46.99%, respectively (Figure 6b). This should be attributed to enhanced intracellular uptake of the developed vesicle due to folate receptors and subsequent rapid intracellular release of Dox due to the sensitivity of the endocytosed vesicles to the high GSH levels present in the cytoplasm of cancer cells. 

**Figure 7:**
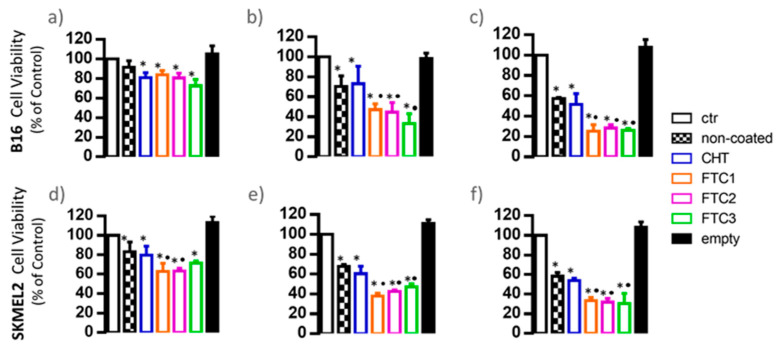
B16-F10 (**a**–**c**) and SKMEL2 (**d**,**f**) cell viability when exposed to empty and Dox-loaded liposomes coated with different polymers after 48 h at Dox concentrations of 1.0 (**a**,**d**) and 3.0 (**b**,**e**) and 6.0 (**c**,**f**) μΜ. Cell proliferation is expressed as mean ± S.D. of three experiments and as the percentage of the control assumed to be 100%. * *p* < 0.05 vs. control; • *p* < 0.05 vs. CHT-coated liposome- treated cells.

The viability of SKMEL2 cells was also assessed, and the process was repeated for B16-F10 cells since an even higher Dox concentration of 6 μΜ was used. As seen, concerning the effects of coated vesicles loaded with 1 and 3 μΜ Dox on the viability of B16-F10 cells (Figure 7a,b), the results of the second study agree with those of the first study (Figure 6b). Indeed, the results obtained in both studies showed an enhancement of the anticancer activity of FTC-coated Dox-loaded liposomes (compared to plain CHT-coated ones) that was achieved only at the higher Dox concentration (3 μM) used, but not at the lowest one (1 μM). 

This could be attributed to the fixed ratio of FTC/Dox in the liposomes, which leads to higher amounts of the new conjugates (with targeting properties) to be available for interaction with the cells only when increased Dox concentrations are used. In other words, it seems that a threshold amount of FTC is necessary to confer measurable targeting properties to the vesicles. 

Regarding the viability of human SKMEL2 cells, it was demonstrated that the vesicles coated with FTC1 and FTC2 have significantly higher anticancer activity even at the low Dox dose of 1 μM (Figure 7d). At the higher Dox doses of 3 μM (Figure 7e) and 6 μM (Figure 7f), all types of FTC-coated Dox-loaded liposomes confer significantly higher reduction in SKMEL2 cell viability, as also observed in the case of B16-F10 cells. Finally, for both cell types, a clear Dox dose response effect on their viability was proven.

From the previous results, we can determine that the amounts of smart multifunctional polymer used to confer targeting properties towards cancer cells are significantly lower than those used in our previous work, and this represents an important advantage of the formulations developed suggesting a higher probability to be translated in a pharmaceutical product. Finally, it was demonstrated that the targeted liposomes had a higher anticancer activity compared to the non-targeted ones. 

This suggests that the developed nanoliposomes could penetrate the cells via endocytic processes and release the drug in the cytoplasm where the GSH level is higher and the disulfide bonds can be broken, facilitating drug release. Our formulations could thus be used in the future as multifunctional devices for controlled release of Dox.

In order to understand to what degree the increased reduction in cell viability by Dox-loaded liposomes that are coated with FTC polymers compared to the ones coated by plain CHT is due to the increased cellular uptake of the various vesicle types, we carried out a cell uptake study. As control liposomes, we used non-coated ones.

**Figure 8:**
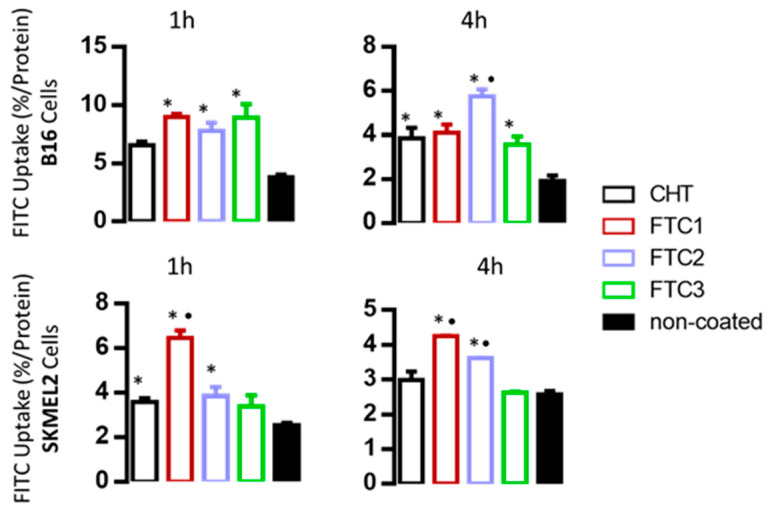
FITC uptake (% Uptake/Protein) by B16-F10 cells (top graphs) and SKMEL2 cells (bottom graphs) following incubation of 106 cells with 400 nmoles (liposomal lipid), non-coated, CHT-coated and with different polymers, at 37 °C for 1 h and 4 h. Results are mean ± S.D. of three experiments * *p* < 0.05 vs. control; • *p* < 0.05 vs. CHT-coated liposomes.

The cell uptake results reported in Figure 8 show that in most cases, an increased uptake of CHT-coated liposomes compared to the non-coated ones is demonstrated; this is observed after 4 h of co-incubation of liposomes with murine (B16 cells) and after 1 h for the human cells, indicating a difference between the two cells with respect to the uptake and release kinetics of the liposomes by the cells. 

The importance of incubation time for the relevant uptake values measured for the different types of vesicles was thus revealed. Although the differences are not significant in some cases, a general remark is that the uptake of FTC1- and FTC2-coated vesicles is higher compared to the uptake of the vesicles coated with plain CHT, which correlates well with the cell viability results of Figure 6 and Figure 7. Nevertheless, similar effects of Dox-loaded liposomes on cell viability (for both cell types evaluated) were demonstrated for FTC3-coated liposomes (compared to the liposomes coated with FTC1 and FTC2 polymers). This may indicate that for FTC3-coated liposomes, either cell uptake is not the main mechanism explaining the increased reduction in cell viability or that perhaps the kinetics of the uptake of the FITC3 vesicles by the cells is different and the specific co-incubation times selected were not ideal. 

Finally, our results agree with a previous report that chitosan coating increases the uptake of indocyanine green loaded liposomes by B16-F10 cells following 4 h co-incubation [44]. 

## 4. Conclusions

In summary, we developed a multitargeted liposomal platform for enhanced intracellular Dox delivery in anticancer therapy. To achieve this goal, novel multifunctional chitosan conjugates combining active targeting and redox responsivity potential were synthetized and used to coat liposomal formulations.

The designed liposomes showed a GSH-dependent drug release profile and enhanced cytotoxicity towards murine and human melanoma cell lines compared to non-target liposomes. Cellular uptake studies demonstrated an increased uptake of CHT-coated vesicles compared to non-coated ones and a further increased uptake of the novel FTC polymer-coated vesicles (compared to the plain CHT-coated ones), in most cases explaining their enhanced cytotoxicity. We believe that physical coating of liposomes with the developed polymers can enhance tumor specificity of nanodevices and offer a promising approach for smart drug delivery. Although future research work is necessary to evaluate to test these liposomal systems in animal models to obtain in vivo data, these early findings are a promising first step for the development of multi-functional devices.

## Figures and Tables

**Table 1:** Amounts of reagents used for the synthesis of TC conjugates with different weight ratios of L-cysteine.

Conjugate	Chitosan (mg)	L-Cysteine (mg)	CMCT (mM)
Thiolated Chitosan 1	150	150	50
Thiolated Chitosan 2	150	300	150
Thiolated Chitosan 3	150	600	150

**Table 2:** The –amount of thiol groups and disulfide bonds grafted on chitosan obtained by the iodine titration method.

Conjugate	CHT/L-Cys/FA Ratio (wt/wt/wt)	-SH Free Total (mol/g Polymer)	-S-S Bond (mol/g Polymer)	Percentage (%) -S-S- Bonds: -SH Free Total
FTC1	1:1:0.1	9.02 × 10^−5^	3.70 × 10^−5^	41.13
FTC2	1:2:0.1	1.22 × 10^−4^	4.1 × 10^−5^	33.61
FTC3	1:4:0.1	1.60 × 10^−4^	9.92 × 10^−5^	62.00

**Table 3:** Physicochemical properties (hydrodynamic diameter, polydispersity index and zeta potential) of empty, calcein and Dox liposomes based on PC/PG/Chol before and after coating with CHT and FTC polymers.

Formulation	Size (nm)	PI	Zeta Potential (mV)
EMPTY LIPOSOMES			
Non-coated	80.10 ± 4.60	0.233	−20.9 ± 1.1
Chit-coated	157.40 ± 31.09	0.349	+29.2 ± 2.1
FTC1-coated	92.58 ± 10.85	0.306	+32.3 ± 2.9
FTC2-coated	120.90 ±9.41	0.222	+27.8 ± 0.4
FTC3-coated	103.60 ± 2.80	0.280	+31.2 ± 01
CALCEIN LIPOSOMES			
Non-coated	57.35 ± 0.78	0.221	−11.5 ± 0.4
Chit-coated	135.20 ± 3.44	0.281	+29.6 ± 3.7
FTC1-coated	104.20 ±1.61	0.334	+38.2 ± 1.0
FTC2-coated	89.37 ± 2.34	0.267	+17.8 ± 3.9
FTC3-coated	83.12 ± 4.65	0.352	+17.7 ± 4.5
DOX LIPOSOMES			
Non-coated	134.10 ± 1.35	0.260	−14.1 ± 1.5
Chit-coated	400.90 ±15.37	0.380	+25.6 ± 4.8
FTC1-coated	162.50 ± 3.93	0.326	+29.5 ± 2.2
FTC2-coated	225.04 ±17.33	0.324	+21.5 ± 0.8
FTC3-coated	159.92 ± 4.07	0.205	+27.9 ± 1.2
FITC LIPOSOMES			
Non-coated	99.43 ± 0.97	0.200	
Chit-coated	150.7 ± 3.85	0.503	
FTC1-coated	160.40 ± 6.50	0.454	
FTC2-coated	143.0 ± 3.39	0.449	

## Data Availability

The data presented in this study are available upon request from the corresponding author.
